# Static glabellar lines treated with the Endolift laser (1470 nm diode laser): A case report

**DOI:** 10.1111/srt.13664

**Published:** 2024-04-01

**Authors:** Mohammad Ali Nilforoushzadeh, Niloufar Najar Nobari, Nazila Heidari, Amirhossein Heidari, Yekta Ghane, Hanieh Azizi, Shohreh Rafiee

**Affiliations:** ^1^ Skin Repair Research Center Shahid Beheshti University of Medical Sciences Tehran Iran; ^2^ Skin and Stem Cell Research Center Tehran University of Medical Sciences Tehran Iran; ^3^ Department of Dermatology Rasool Akram Medical Complex Clinical Research Development Center (RCRDC) School of Medicine Iran University of Medical Sciences Tehran Iran; ^4^ School of Medicine Iran University of Medical Sciences Tehran Iran; ^5^ Faculty of Medicine Tehran Medical Sciences Islamic Azad University Tehran Iran; ^6^ School of Medicine Tehran University of Medical Sciences Tehran Iran

Dear Editor,

Wrinkles are obvious lines or folds in the skin that make an inappropriate appearance on the face and negatively affect mental health and self‐esteem. Glabellar lines, also known as worry lines, are caused by contractions of different muscles of the face, including the corrugator, depressor supercilii, and/or procerus muscles, leading to an undesirable gap between the eyebrows.^1^ Several procedures, such as surgery, botulinum toxin type A injection, and implants, have been utilized for the treatment of wrinkles, although patients usually prefer non‐invasive and minimally invasive methods.^2^ Herein, we reported a patient with deep glabellar lines successfully treated with Endolift, a minimally invasive approach.

A 42‐year‐old female suffering from vertical glabellar lines was presented to Jordan Dermatology and Hair Transplantation Center, Tehran, Iran. Over the past year, she underwent botulinum toxin type A injection twice and filler injection once, which all failed to improve her vertical glabellar lines (Figure 1). Before the treatment, the targeted area was carefully examined and the subject was informed about the contraindications, warnings, and possible complications associated with the Endolift. The area was cleaned up using a remover solution and the patient was positioned on a flatbed. After receiving injectable local anesthesia with lidocaine, she was treated with one session of Endolift (LASEMAR1500 machine, Eufoton s.r.l.) with the setting of power of 3.5–4 WATTs, total energy of 150 J, T ON of 25 ms and T OFF of 75 ms, and fiber of 400µm. The size of the fiber was adjusted based on the depth of the wrinkle and the thickness of the skin. The fiber was transferred vertically under the glabellar line with reciprocal motion in the targeted area. The technique did not require recovery time. Regular photography (with a Nikon 10.2‐megapixel camera) was taken of the patient before and after the procedure as well as one month after treatment. After the procedure, a remarkable decrease in the depth of the glabellar lines and muscle bulk of the treated area was observed (Figure 2). Moreover, the patient's satisfaction was evaluated based on a four‐point scale from very satisfied to unsatisfied (from four to one, respectively) As a result, the subject was very satisfied after a single session of treatment. Regarding adverse events, the patient reported mild pain, redness, and swelling in the treatment area, which resolved after a few days. Subsequently, four weeks after the procedure, the patient was very satisfied with the treatment. Moreover, a remarkable improvement was reported at a follow‐up visit by a dermatologist (Figure 3).

**FIGURE 1:**
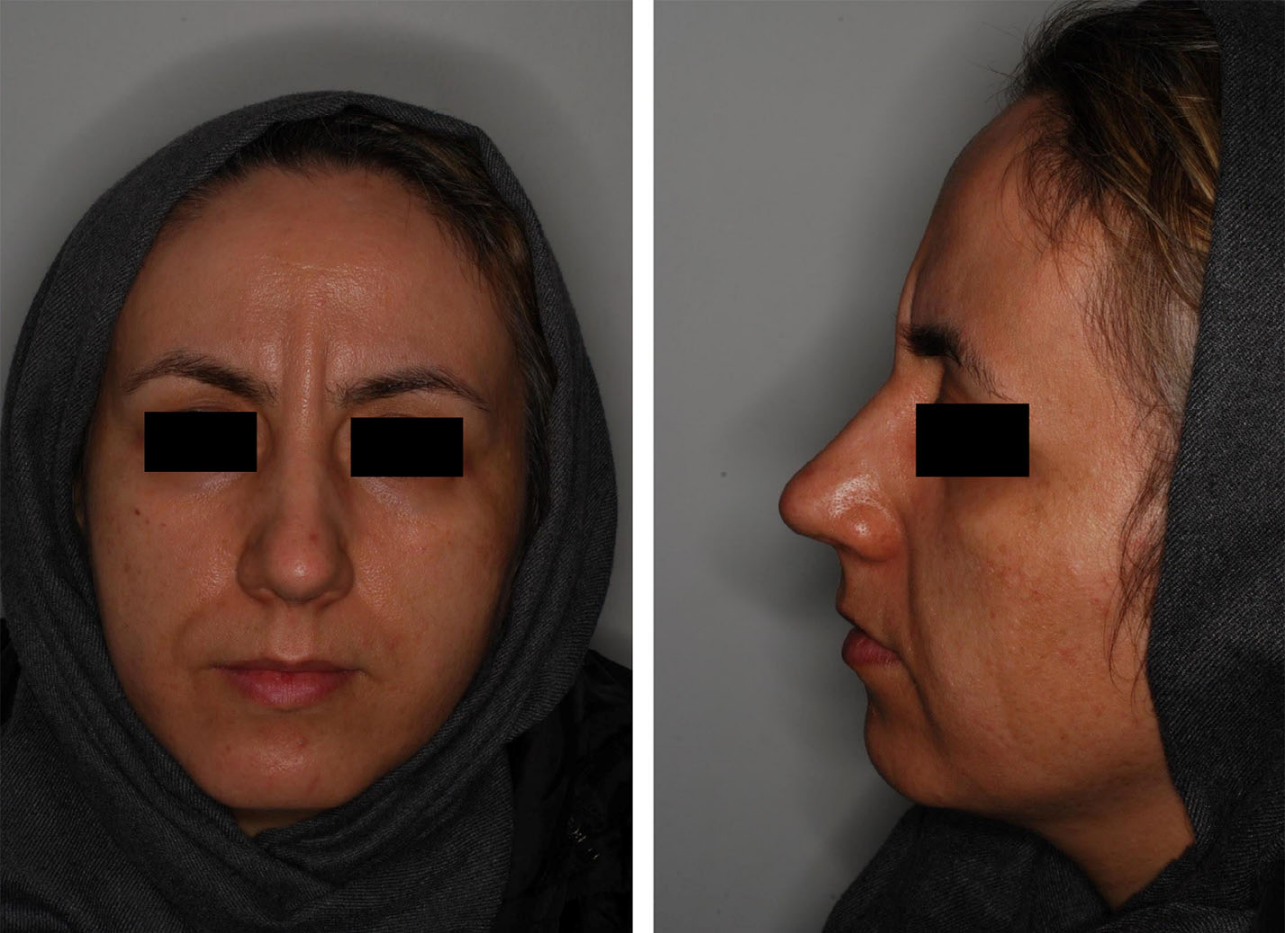
The patients' glabellar lines during the first visit before the Endoift treatment.

**FIGURE 2:**
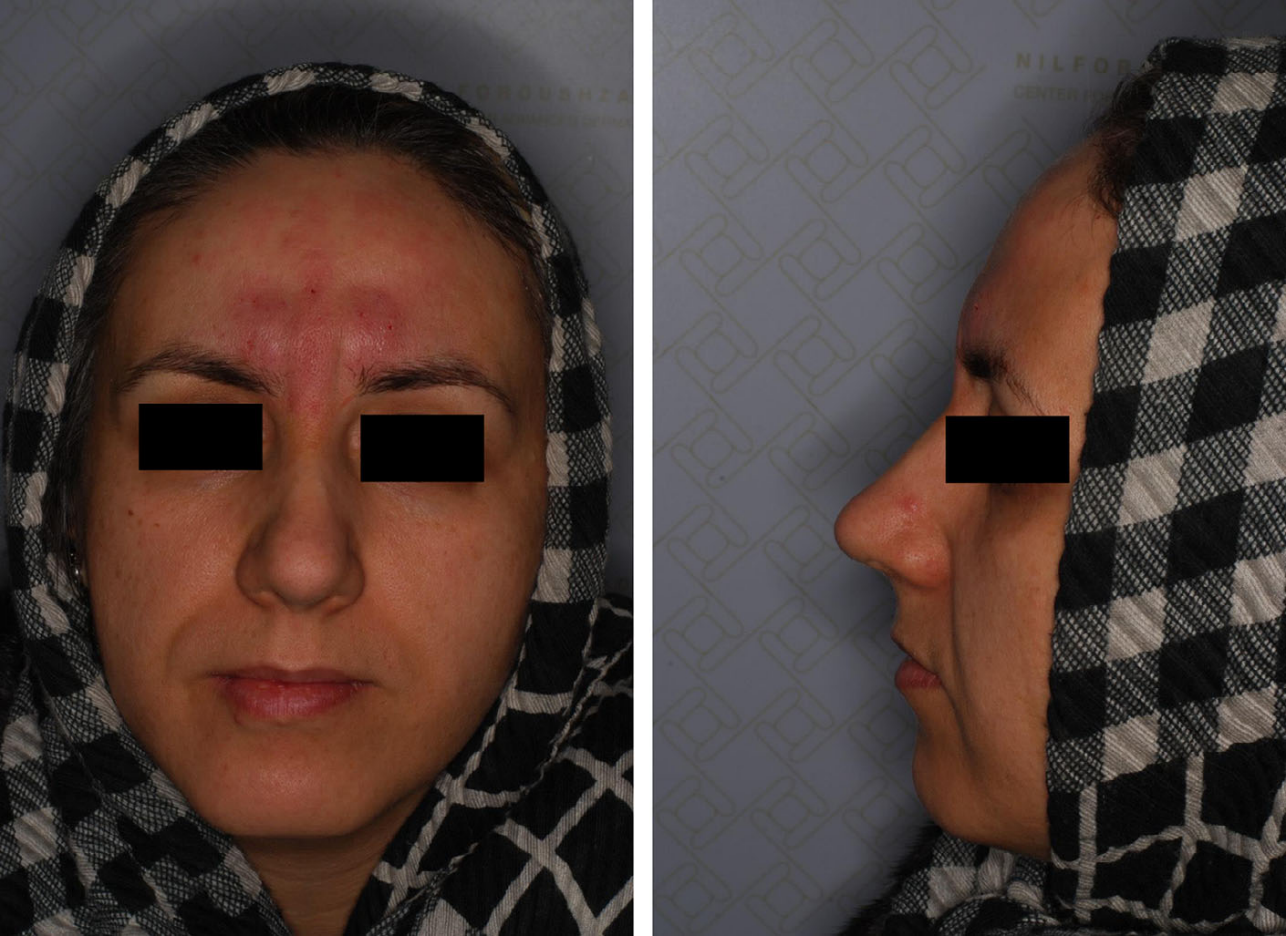
The patient immediately after the Endolift procedure for the treatment of glabellar lines.

**FIGURE 3:**
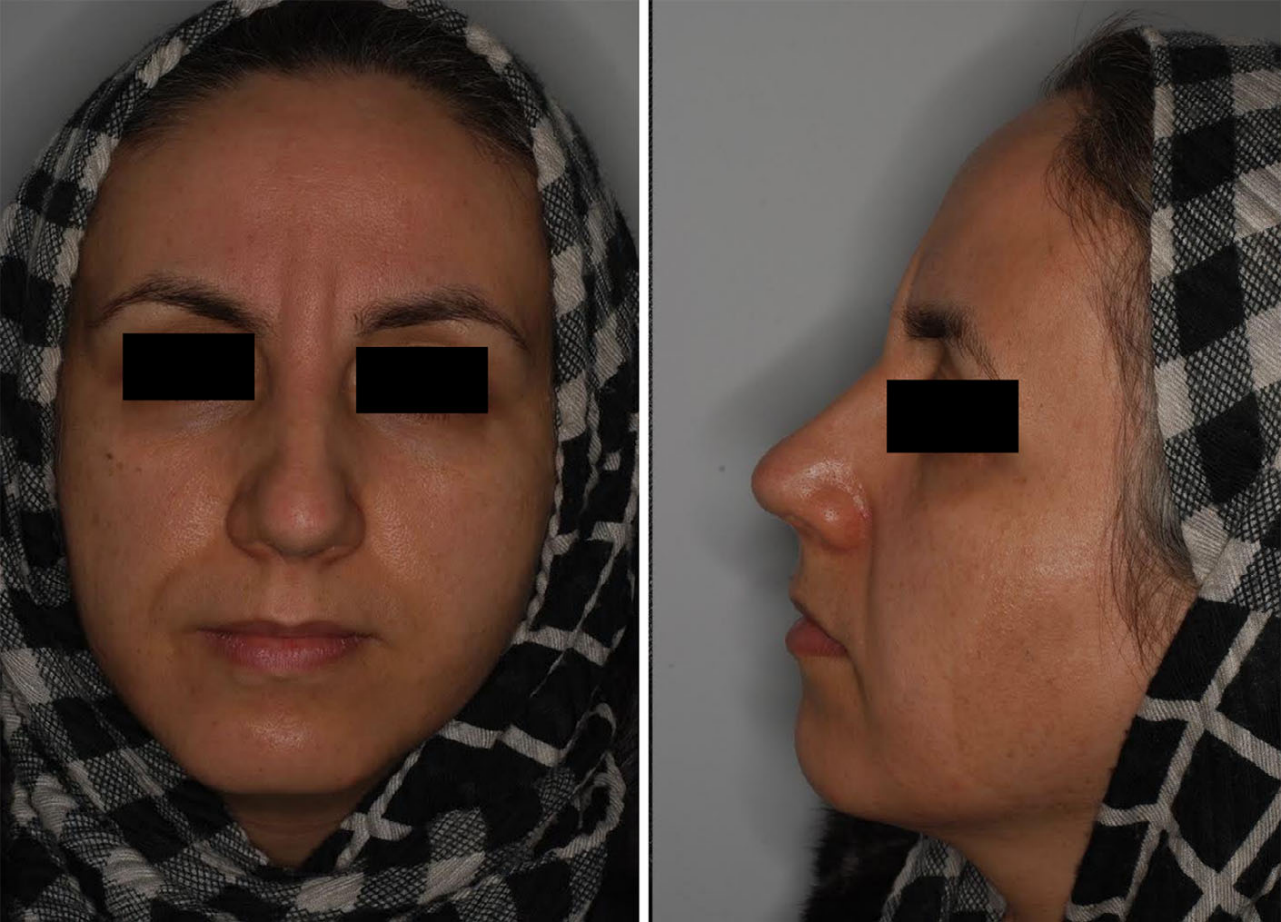
Four weeks after the Endolift procedure for the treatment of glabellar lines.

Various therapeutic approaches have been utilized for the improvement of wrinkles. Previously, surgical procedures, such as the endoscopic facelift technique, were the most frequent rejuvenation procedures with acceptable results.^3^ Nonetheless, in some cases, surgical procedures aggravated the volume loss. Besides, they increase the risk of forehead area nerve and blood vessel damage and hairline alteration.^4^ Botulinum toxin type A is a popular, easy treatment technique for the resolution of upper face area rhytides with desirable but reversible effects, which needs repeated rounds of injection to maintain the result.^5^ Radiofrequency is another potential therapeutic choice with the ability to enhance collagen synthesis and dermal remodeling; however, it may cause scarring, depigmentation, and eruptive keratoacanthomas.^6^

Endolift laser is a novel, minimally invasive method that has been utilized in various cosmetic fields with desirable outcomes^5^. This procedure uses a non‐ablative fractional diode laser with a wavelength of 1470 nm as the energy source and transmits the energy to the treated tissue via a fiber 200–600 µm in diameter, eventually providing focused tissue ablation and coagulation. Aside from its clinical effectiveness, Endolift does not cause adverse events such as scarring, muscle atrophy, brow ptosis, and facial nerve damage, which commonly occur following surgery and botulinum toxin type A injection. Furthermore, no recovery time is needed for this procedure, and patients are able to go back to their daily routine immediately. Nonetheless, large‐scale randomized controlled trials are warranted to assess the efficacy and safety of this procedure and its different implications.

The application of Endolift was successful in treating glabellar lines. Endolift is a safe and effective method for promoting skin rejuvenation as well as improving facial wrinkles and the overall aesthetic appearance of the face with self‐limited adverse events. However, further investigations should be conducted to evaluate the efficacy and safety of this rejuvenation method.

## CONFLICT OF INTEREST STATEMENT

The authors declared no conflict of interest.

## ETHICS APPROVAL AND CONSENT TO PARTICIPATE

All procedures followed were in accordance with the ethical standards of the responsible committee on human experimentation (institutional and national) and with the Helsinki Declaration of 1975, as revised in 2000. Formal consent is not required for this type of study.

## CONSENT FOR PUBLICATION

Written informed consent was obtained from the patient for publication of this case report and any accompanying images. A copy of the written consent is available for review by the Editor‐in‐Chief of this journal.

## Data Availability

Our data included personal patient data. Additional data are available from the corresponding author upon reasonable request.
